# Drought-Tolerant Corn Hybrids Yield More in Drought-Stressed Environments with No Penalty in Non-stressed Environments

**DOI:** 10.3389/fpls.2016.01534

**Published:** 2016-10-13

**Authors:** Eric Adee, Kraig Roozeboom, Guillermo R. Balboa, Alan Schlegel, Ignacio A. Ciampitti

**Affiliations:** Throckmorton Plant Science Center, Department of Agronomy, Kansas State University, ManhattanKS, USA

**Keywords:** corn, drought tolerant, yield, genotype, management, environment

## Abstract

The potential benefit of drought-tolerant (DT) corn (*Zea mays* L.) hybrids may depend on drought intensity, duration, crop growth stage (timing), and the array of drought tolerance mechanisms present in selected hybrids. We hypothesized that corn hybrids containing DT traits would produce more consistent yields compared to non-DT hybrids in the presence of drought stress. The objective of this study was to define types of production environments where DT hybrids have a yield advantage compared to non-DT hybrids. Drought tolerant and non-DT hybrid pairs of similar maturity were planted in six site-years with different soil types, seasonal evapotranspiration (ET), and vapor pressure deficit (VPD), representing a range of macro-environments. Irrigation regimes and seeding rates were used to create several micro-environments within each macro-environment. Hybrid response to the range of macro and micro-environmental stresses were characterized in terms of water use efficiency, grain yield, and environmental index. Yield advantage of DT hybrids was positively correlated with environment ET and VPD. Drought tolerant hybrids yielded 5 to 7% more than non-DT hybrids in high and medium ET environments (>430 mm ET), corresponding to seasonal VPD greater than 1200 Pa. Environmental index analysis confirmed that DT hybrids were superior in stressful environments. Yield advantage for DT hybrids appeared as yield dropped below 10.8 Mg ha^-1^ and averaged as much as 0.6–1 Mg ha^-1^ at the low yield range. Hybrids with DT technology can offer a degree of buffering against drought stress by minimizing yield reduction, but also maintaining a comparable yield potential in high yielding environments. Further studies should focus on the physiological mechanisms presented in the commercially available corn drought tolerant hybrids.

## Introduction

In agriculture, drought can be defined by the absence of soil water to provide conditions for crops to grow as a consequence of precipitation being less than normal (UNISDR, 2009). In recent years, several research projects have been conducted to discover physiological mechanisms to increase crop performance under drought. Improving tolerance to drought has become increasingly important for corn (*Zea mays* L.) production for several reasons (Chen et al., 2012). In the traditional Corn Belt of the U.S., periods of drought at critical growth stages can negatively impact yield even if soil moisture is not limiting at other stages of development (Roth et al., 2013). Lobell et al. (2014) suggested that historic corn yield gains have been coupled with increasing planting densities in the U.S. and were accompanied by increased sensitivity to drought in modern hybrids. As corn acres have increased outside of the Corn Belt region, drought tolerance has become an even more important trait for corn. Typically these non-traditional areas do not have the soil moisture holding capacity and/or the rainfall typical of the Corn Belt region. As an example of the impact of drought on corn yield, the 2012 drought in the U.S. decreased grain yield by 21% compared to the previous 5 years with an average country-yield of 7.7 mg ha^-1^ (Boyer et al., 2013).

Seed companies have begun to release corn hybrids classified as drought-tolerant (DT) to provide protection from occasional drought stress in areas where corn has traditionally been grown and to enhance the viability of corn in areas prone to drought stress. Three corn hybrid technologies currently being marketed for DT include Pioneer Optimum AQUAmax^TM^ (DuPont Pioneer, Johnston, IA, USA) and Syngenta Artesian^TM^ (Syngenta Seeds, Minnetonka, MN, USA), both promoted as achieving drought tolerance through traditional breeding. The third drought tolerant technology is Monsanto’s Genuity^TM^ DroughtGard^TM^ (Monsanto Co., St. Louis, MO, USA), promoted as conferring drought tolerance through both traditional plant breeding and the introduction of a transgenic trait. The transgenic trait results in the expression of bacterial cold shock protein B (Nemali et al., 2015). Cold shock proteins contain RNA binding sequences referred to as cold shock domains and are well known to act as RNA chaperones (Horn et al., 2007; Nemali et al., 2015). A number of native corn traits associated with greater drought tolerance have been targeted, e.g., reduced anthesis-silking interval, improved dry matter and nutrient partitioning, and robust root systems. Messina et al. (2015) demonstrated that hybrids expressing the limited transpiration trait increased yields when drought stress occurred at flowering and during grain-fill. Limited transpiration could be associated with differences in plant structure, such as a more extensive root system, narrower leaves, or fewer stomata, or it could be more physiological in nature such as modified stomatal regulation. Regardless of the mechanisms conferring drought tolerance, yield potential is the main priority when farmers select hybrids. Therefore, characterizing the yielding ability of DT hybrids relative to that of non-DT hybrids in a wide range of yield environments is critical for hybrid selection, especially for environments with variable water supply from year to year.

Determining if there are environments that favor DT hybrids and if DT hybrids are more or less sensitive to plant density would be critical information for the placement and management of these hybrids for the greatest benefit. Roth et al. (2013) evaluated one DT hybrid and one non-DT hybrid in 2011 (a normal year) and 2012 (severe drought) with similar responses in grain yield, leaf photosynthesis, and leaf transpiration for both hybrids. Carretero et al. (2014) established that hybrids with greater root density explore deeper soil layers, providing a strategy to tolerate water stress in rainfed conditions. In water limited environments (U.S. Western Corn Belt) during 3 years of testing, Gaffney et al. (2015) reported a 4.9% increase in yield for DT hybrids compared to non-DT hybrids in 53 water-limited environments and a 2.5% yield increase in 502 water-favorable environments from small-plot studies. The same authors reported a 6.5% yield advantage for DT hybrids evaluated in a total of 2006 water limited environments and a 1.9% yield advantage in 8725 water-favorable environments from on-farm strip trials conducted during 2011 to 2013.

Questions remain regarding how best to utilize these DT hybrids. The first question regards the yield potential of DT hybrids relative to non-DT hybrids. This is especially important for fields that have variable yield potential within a year or from year to year. Knowing the yield potential of DT hybrids relative to non-DT hybrids in favorable yield conditions would be critical information for growers to have when selecting hybrids. If growers know they are giving up yield potential to gain tolerance to occasional drought, they would have to weigh the benefits and risks when selecting hybrids. If there is no difference in yield potential between the DT and non-DT hybrids, then selection of hybrids would be simplified. This leads to a second question: How do yields of DT hybrids compare to non-DT hybrids in environments with different yield potential? Determining if there are environments that favor DT hybrids is critical information for the proper placement of DT hybrids to realize the greatest benefit from this genetic technology. A third question arises regarding the relative WUE of DT and non-DT hybrids. This information would help better understand why DT hybrids may have an advantage in certain environments.

Additional analysis of the existing information about yields of DT and non-DT hybrids can improve our understanding of their behavior at varying yield levels. DeWitt and Langerhans (2004) highlight that phenotypic plasticity is one of the strategies used by plants to respond to environmental variation. Bradshaw (1965) defined phenotypic plasticity as “the amount by which the expressions of individual characteristics of a genotype are changed by different environments.” In this paper, the concept of phenotypic plasticity is also referred to as environmental index. Analysis of environmental index can make effective use of a large yield database for better understanding the behavior of DT and non-DT hybrids across the large range of environments where these hybrids are likely to be deployed. The slope for the yield and environmental index relationship refers to the yield adaptability for each specific hybrid technology evaluated. Slopes <1 indicate that the hybrids in question have more stable yields across different environmental indexes, and slopes >1 indicate that the hybrids have less yield adaptability, changing yields as the environmental index varies. Additionally, comparisons of the WUE between DT and non-DT hybrids could help explain any differences between the hybrids and assist in decisions on where they should be planted. We hypothesized that corn hybrids containing DT traits would produce more consistent yields compared to non-DT hybrids in the presence of plant stress due to combinations of reduced water supply and increased plant density. The primary objective of this study was to compare the response of DT with non-DT hybrids in environments representing a wide range of yield potential due to drought stress.

## Materials and Methods

Drought tolerant and non-DT hybrid pairs of similar maturity were planted in locations that varied in soil characteristics and seasonal ET to generate a range of macro-environments differing in the likelihood and severity of drought stress. Irrigation (IRRI) regimes (**Table 1**) and seeding rates (SRs) were used to create different levels of stress within each macro-environment.

**Table 1:** Parameters for crop ET, precipitation, and irrigation from KanSched2 Irrigation Program, soil type, and corn yield at six site-years: Scandia, 2012, 2013; Topeka, 2012, 2013; Hutchinson, 2013; and Tribune, 2013.

Environment	High ET (>508 mm)				Medium ET (from 432 to 488 mm)				Low ET (<432 mm)			
Site-year	Tribune, 2013		Topeka, 2012		Hutchinson, 2013		Scandia, 2012		Scandia, 2013		Topeka, 2013	
Soil series†	Ulysses silt loam		Eudora silt loam to sandy loam		Nalim loam		Crete silt loam		Crete silt loam		Eudora silt loam to sandy loam	
SWH capacity‡ (mm)	2824		777		937		1059		1059		777	
Planting date	6-May		17-April		14-May		22-May		30-April		30-April	
Irrigation regime (%ET)	100	50	100	50	100	50	100	50	100	50	100	50

Crop ET^§^ (mm)	760	579	559	447	488	422	483	483	401	386	417	356

Seasonal Vapor Pressure Deficit (Pa)^∗^	1261		1375		973		1325		972		924	
Precipitation (mm)	404	404	165	165	615	615	203	203	224	224	358	358
Irrigation (mm)	414	193	305	173	102	51	254	127	140	67	229	58
Grain Yield^#^ (Mg ha^-1^)	13.8	10.4	10.9	9.2	9.7	10.5	12.4	7.8	11.5	10.7	14.2	10.3

*Sites were grouped by similar ET values relative to each other, and classified as high, medium, and low. †From USDA-NRCS Web Soil Survey (http://websoilsurvey.nrcs.usda.gov/app/). ‡Estimated from National Engineering Handbook Part 652, Chapter 2: http://www.nrcs.usda.gov/Internet/FSE_DOCUMENTS/nrcs142p2_031591.pdf^§^Estimated by KanSched for the full irrigation scenario at each location based on reference ET modified by crop coefficients appropriate to vegetative growth, reproductive stage, and senescence growth stages. The ET estimation is across all hybrid by seeding rate treatments within an irrigation regime and does not reflect the ET for individual hybrid by seeding rate treatments. ^∗^Vapor pressure deficit (VPD) = ((100 - RH)/100)^∗^saturated vapor pressure (SVP), SVP (Pascals) = 610.7^∗^10*^*7.5 T/(237.3+T)*^*, Temperature (T). ^#^Grain yield is an average of hybrid by seeding rate treatments within an irrigation regime.*

A total of six experiments were conducted at four locations in Kansas: Kansas River Valley Experiment Field at Topeka (39° 4′36.84″N, 95°46′5.68″W) and North Central Experiment Field at Scandia (39°49′54.56″N, 97°50′23.28″W) in 2012 and 2013; Southwest Research Center at Tribune (38°31.7′ N, 101°39.7′ W), and South Central Experiment Field, Hutchinson (37°55′51.01″N, 98° 1′42.42″W) in 2013. The sites were selected based on the total amount of precipitation and variation in crop ET. Further details related to site differences are presented in **Table 1**.

Two pairs of DT and non-DT hybrids, with similar maturity, were selected as considered being well adapted at each site. At Topeka and Scandia sites the hybrid pairs used in 2012 were Pioneer (Pioneer Hi-bred, Johnston, IA, USA) P1151 Aquamax (DT, 111 RM) and P1162 (non-DT, 111 RM), and P1498 Aquamax (DT, 114 RM) and P33D49 (non-DT, 115 RM). In 2013 at Hutchinson and Topeka sites, the hybrid pairs were Pioneer P1151 Aquamax and P0987 (non-DT, 109 RM) and DeKalb (Monsanto, St. Louis, MO, USA) DKC63-55 DroughtGard (DT, 113 RM) and DKC63-84 (non-DT, 113 RM), and at Tribune and Scandia, the hybrid pairs were Pioneer P1151 Aquamax and P0987 and DeKalb DKC62-26 DroughtGard (DT, 112 RM) and DKC63-07 (non-DT, 113 RM). The sub-plots in all experiments were 3 m wide by 10–14 m long in four 0.76 m wide rows at four SRs varying from 61,750 to 98,800 seed ha^-1^ in 12,350 increments. Experiments at Topeka, Hutchinson, and Tribune were planted at the four SRs, and stand counts confirmed that final stands were within 95% of the target plant density (data not shown). The experiments at Scandia were planted at a uniformly high density and thinned to three plant densities in 2012 (74,100, 86,000, and 98,800 plants ha^-1^) due to space constraints and all four densities in 2013.

The fertility was maintained at optimum for each location, and pre- and post-emerge herbicides were applied at recommended rates to eliminate any confounding factors caused by weeds. All hybrids were protected against corn rootworm (*Diabrotica* species), and European corn borer, *Ostrinia nubilalis* (Hubner) utilizing the respective Bt genes. Fungicide applications were not needed because disease severity did not reach economic thresholds for application.

At each location, IRRI regimes of 50 and 100% ET were applied uniformly across all combinations of hybrids and plant densities utilizing sprinkler IRRI systems. IRRI amounts and timing were based on the 100% ET regime estimated using the KansSched2 IRRI scheduling program (Rogers and Alam, 2008). The KansSched2 program utilizes an algorithm which incorporates crop growth stage and maturity length, daily ET values, calculated soil profile moisture, rainfall, and IRRI from each location. This algorithm does not include SRs or water use of specific hybrids. The 50% IRRI regime was achieved by either irrigating every other pass or adjusting the number of nozzles to apply half as much water as the 100% IRRI regime. IRRI regimes of 50 and 100% ET were implemented at all locations. Daily ET values and rainfall data for each site were obtained from the Weather Data Library at Kansas State Agronomy (Weather Data Library, 2016) for each location. The Maximum Allowable Deficit (MAD) (Rogers and Alam, 2008) is defined as the maximum percent of available soil water removed by the crop before IRRI is needed; set at 50% for this study. The soil moisture with the 50% IRRI regime dropped below MAD just before or just after tasseling at all environments. The number of days of moisture stress varied by site, dependent on rainfall distribution. One of the most stressed environments was at Topeka in 2012, with the soil moisture continuously below MAD from tasseling until crop maturity under the 50% ET IRRI regime. In contrast, at Hutchinson in 2013, the soil moisture was below MAD only 18 out of 51 days after tasseling in the 50% ET IRRI regime.

At all locations except Hutchinson, the experiment was arranged in a randomized complete-block design (RCBD) with a split plot treatment structure. IRRI regime was the whole plot, and combinations of hybrid and SR were allocated to the sub-plots. There were three or four replications of all treatment combinations at each site. The macro-environment at Hutchinson in 2013 differed in that IRRI was not a randomized treatment effect, but was simply another environment containing the four replications of all combinations of hybrid and SR. Due to the difference in the design of the study, the data from Hutchinson was not combined with data from another site with similar ET, but analyzed and presented separately. For terminology purposes, the term micro-environment will refer to sub-plot, primarily based on the SR by hybrid combination, at each specific IRRI level within each site.

Soil moisture was measured to the effective rooting depth at three macro-environments: Scandia (2012, 2013) (Gordon et al., 1995); and Tribune (2013). Soil water content was measured by neutron thermalization with a 503 DR Hydroprobe Moisture Gauge (CPN International, Inc., Martinez, CA, USA) using a count duration of 16 s. Access tubes of standard type 6061-T6 aluminum tubing (o.d. 4.128 cm, wall thickness 0.089 cm) 1.15 m in length were installed in the field plots to a depth of 1.0 m at Scandia in all plots. Tubes were 2.6 m in length and installed to a depth of 2.4 m at Tribune in plots with the high and low SRs for all hybrids in both IRRI regimes. Starting at a depth of 0.152 m below the soil surface, water content was measured in 0.305 m increments. There was no seal at the base of tubes, and tubes were capped between measurements made at the beginning of the season, shortly after planting, and at the end of the season soon after the crop had reached black-layer.

### Drought Tolerant (DT) versus non-DT Analysis

The predicted yield advantage of DT hybrids over the non-DT hybrid across the range of micro-environments generated in this study was modeled by a fitting segmented model using PROC NLIN in SAS 9.4 (SAS Institute, 2009). Yield potential of each micro-environment was characterized by the mean yield of the non-DT hybrid in that micro-environment (*n* = 3 or 4). Yield advantage of the DT hybrid in a given micro-environment, calculated as mean DT hybrid yield minus mean non-DT hybrid yield, was regressed on mean non-DT hybrid yield in an iterative process that minimized model sums of squares. Conditions were imposed to assure a continuous, smooth transition from a linear segment to a plateau segment with an unknown point of coincidence.

### Environmental Index

In order to relate the response of different phenotypes to the different environments, grain yield of DT and non-DT hybrids were regressed on yield means for each micro-environment (combination of location, IRRI regime, SR, and hybrid, *n* = 54) to characterize their response to environmental yield potential. The slope of this mathematical function is a measure of yield adaptability (Finlay and Wilkinson, 1963; Dingemanse et al., 2010). Slopes >1 indicate a greater change in yield with changing environments, and slopes <1 indicate more stable yields across a range of environments. The environmental yield gradient was generated by the micro-environments resulting from combinations of location, IRRI regime, and SR, *N* = 54.

### Grain Yield and Water Use Efficiency Analyses

Yields were calculated from grain harvested from the center two rows of each four-row plot, and corrected to 15.5% grain moisture. Yield data from each location were subjected to ANOVA using SAS PROC GLIMMIX (SAS Institute, 2009) including a spatial covariate to account for within-replication gradients due to soil variability at all sites (Yang and Juskiw, 2011). For the RCBD studies with a split plot arrangement, sites, IRRI, hybrid, and SR were utilized as fixed factors, and blocks were treated as a random effect. Crop ET as estimated by KansSched2 for the full IRRI scenario was used to group the six site-years into three sets, each containing two environments (Low ET, Medium ET, and High ET), each with similar overall water demand: (1) low ET < 432 mm (Topeka and Scandia, 2013); (2) medium ET from 432 to 488 mm (Scandia, 2012; Hutchinson, 2013), and (3) high ET > 508 mm (Topeka, 2012; Tribune, 2013). Data within the High and Low ET macro-environments were pooled to test for interactions of treatment effects with macro-environment; however, the Medium ET environments had different experimental designs and could not be combined for ANOVA. Yield data from the three sites that measured water use and determined WUE (grain yield to water use ratio) were paired with macro-environments that had similar ET for analysis.

## Results

### Yield Comparison

Averaged across SRs, grain yield ranged from 7.8 (50% ET IRRI, Scandia, 2012) to 14.2 Mg ha^-1^ (100% ET IRRI, Topeka, 2013) (**Table 1**). The three-way interaction, IRRI × hybrid type (HT) × SR, significantly influenced yields in the low ET sites (Topeka and Scandia, 2013) (**Table 2**), implying a possible differential response of the different HTs depending on micro-environment, although the lack of significance for the interaction of HT and SR may indicate that the differential was not necessarily induced by differential response of drought tolerance to SR. In addition, the main difference on the three-way interaction was observed on the 50% ET IRRI with minor yield variations for HT but similar responses to SR within each HT, whereas for the 100% ET yields did not statistically differ regardless of the SR and IRRI type (averaging 12.9 Mg ha^-1^). The two-way interaction of SR and IRRI affected yield at the High ET environments (Tribune, 2013 and Topeka, 2012) but not at the Medium ET environments (Hutchinson, 2013 and Scandia, 2012) and Low ET environments (Scandia, 2013 and Topeka, 2013). SR affected yields in all three ET environment groups (**Table 2**). HT affected yield independent of IRRI regime in High ET environments, but the response was modified by IRRI regime in Medium and Low ET environments (**Table 2**). IRRI regime affected yield at all sites except for Hutchinson in 2013, one of the Medium ET environments.

**Table 2:** Significance (*P* values) of fixed effects for irrigation level (IRRI), hybrid type (HT), seeding rate (SR) and their interactions at high, medium, and low ET (evapotranspiration) sites.

	**High ET (>508 mm)**	**Medium ET (from 432 to 488 mm)**		**Low ET (<432 mm)**
**Source**	**Topeka, 2012; Tribune, 2013**	**Hutchinson, 2013†**	**Scandia, 2012**	**Scandia, 2013; Topeka, 2013**
	***P-*value**			
IRRI	0.002	0.835	<0.0001	0.003
HT	0.003	<0.0001	<0.0001	0.579
HT × IRRI	0.463	0.005	0.0638	0.314
SR	0.025	0.030	<0.0001	0.006
SR × IRRI	<0.0001	0.649	0.484	0.204
HT × SR	0.611	0.082	0.657	0.857
IRRI × HT × SR	0.585	0.823	0.451	0.028

*†Tests of irrigation (IRRI) and its interactions were conducted using conservative error degrees of freedom because irrigation was not replicated.*

Yield advantages were greater for DT over non-DT hybrids in the High and Medium ET environments, but not in the Low ET environments (**Tables 2** and **3**). The yield advantage for the DT over non-DT hybrids in the High and Medium ET environments was approximately 5 to 7% when averaged over all the SRs and IRRI factors (**Table 3**). In the Low ET environment there was no yield difference between the DT and non-DT hybrids when averaged over all the SRs and IRRI factors (**Table 3**).

**Table 3:** Yield for DT and non-DT corn hybrids, yield advantage in absolute and relative terms averaged across plant densities at different macro-environments based on cropping season ET values.

	Macro-environments							
	High ET		Medium ET				Low ET	
	Topeka, 2012; Tribune, 2013		Hutchinson, 2013		Scandia, 2012		Scandia, 2013; Topeka, 2013	
Irrigation regime (ET%)	100	50	100	50	100	50	100	50

	**Grain yields, expressed in Mg ha^-1^**							
DT	13.0	9.2	10.9a†	10.6a	11.0	9.7	12.8	10.6
Non-DT	12.6	8.6	10.0b	10.3b	10.8	8.7	12.9	10.4

DT	11.1a‡		10.8a		10.4a		11.7a	
Non-DT	10.6b		10.1b		9.7b		11.6a	

Yield Adv. for DT	0.53 (5.0%)		0.63 (6.2%)		0.66 (6.7%)		0.08 (0.7%)	

*†Means within an irrigation regime at Hutchinson, 2013 followed by the same letter are not different (*P* < 0.05). ‡Means within a column followed by the same letter are not different (*P* < 0.05).*

### Water Use Efficiency

Analysis of variance revealed a significant effect of HT but no significant effect of seeding density or interactions of HT with seeding density. In the three environments where water use was measured, WUE increased as seasonal ET decreased (**Table 4**). The WUE advantage of DT compared to non-DT hybrids was different depending on the level of ET for the season. The DT hybrids had greater WUE, producing more yield for a given amount of moisture in the High and Medium ET environments (**Table 4**). For both High and Medium ET environments, averaged across the micro-environment factors, grain yield of DT hybrids averaged 1.3 kg ha^-1^ mm^-1^ more than the non-DT hybrids. However, there was no difference in WUE between the DT and non-DT hybrids in the Low ET environment (**Table 4**). The High ET environments produce less kg of grain per unit of water because a greater portion of the water is lost through evaporation from the soil or leaf surfaces, and through transpiration as the plant tries to cool itself. In contrast with the Low ET environments, the efficiency of corn turning water into grain is increased.

**Table 4:** Water Use Efficiency (WUE) (grain kg ha^-1^ mm^-1^of water) for drought tolerant (DT) and regular (non-DT) corn hybrids at three site years: Tribune, 2013 (high and low populations for all hybrids and irrigation regimes); and Scandia, 2012, 2013 (all plots).

Hybrid type	High ET (Tribune, 2013)†	Medium ET (Scandia, 2012)†	Low ET (Scandia, 2013)†
	**WUE (grain kg ha^-1^ mm^-1^ of water)**		
DT	17.2	23.9	32.1
Non-DT	16.4	22.2	32.1

DT WUE Advantage	0.82	1.77	0.05
*P*>*F*	0.01	<0.0001	0.66

*†Only significant results presented. Seeding rate, irrigation regime, and interactions were not significant.*

**FIGURE 1:**
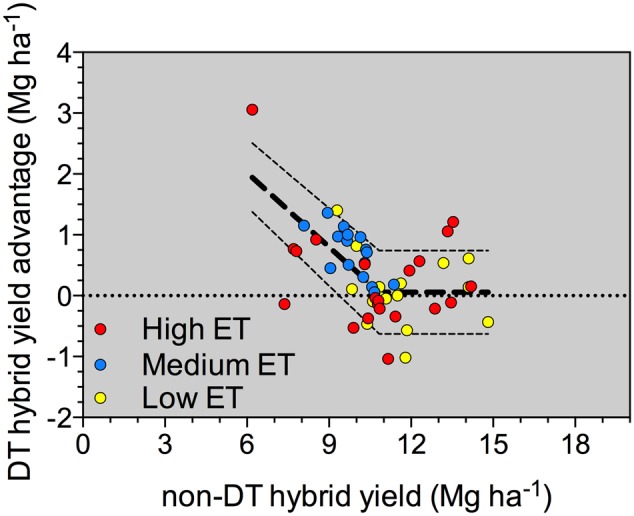
**Yield comparison of DT and non-DT hybrid pairs in high (red circles), medium (blue circles), and low (yellow circles) evapotranspiration (ET) environments across several site-years in Kansas for the 2012 and 2013 growing seasons (*n* = 53).** Model: If NDTYLD < 10.82 Mg ha^-1^, DTYA = 4.45 (+0.41) - 0.405 (+0.0257) × NDTYLD. If NDTYLD ≥ 10.82 Mg ha^-1^; DTYA = 0.051 (+0.68) Mg ha^-1^ (+ indicates 95% confidence interval for each parameter); where NDTYLD = non-DT hybrid yield; DTYA = DT Hybrid Yield Advantage; Model probability of greater *F* < 0.0001; and *r*^2^ = 0.36; RMSE = 0.56.

Yield comparison between DT and non-DT hybrids pairs in all environments are shown in **Figure 1**, adjusted by a linear-plateau model. In the range of micro-environments sampled in this study, if yield of the non-DT hybrid was equal to or greater than about 10.8 Mg ha^-1^, the DT hybrid had a 0.057 Mg ha^-1^ or 57 kg ha^-1^ yield advantage, essentially equal on average. However, if yield of the non-DT hybrid was less than 10.8 Mg ha^-1^, the yield advantage of the DT hybrid increased by an average of 0.41 kg ha^-1^ for every kg ha^-1^ decrease in yield of the non-DT hybrid. Although there was considerable variability in the data set, this model was highly significant and accounted for more variability (*R*^2^ = 0.36) in the data than segmented quadratic-plateau or non-segmented linear or non-linear models. Common sense and the standard precaution of not extrapolating beyond the range of values used to generate this model imply that the yield advantage for the DT hybrid is not likely to continue to increase as yield of the non-DT hybrid continues to decline. However, the model indicates that no consistent yield penalty was evident for the DT hybrid in environments where yields approached 15.7 Mg ha^-1^. A yield advantage for the DT versus the non-DT hybrid appeared, as yield became less than 10.8 Mg ha^-1^ and became consistently greater as yield of the non-DT hybrid dropped to 6.3 Mg ha^-1^ (**Figure 1**).

### Environmental Index

**FIGURE 2:**
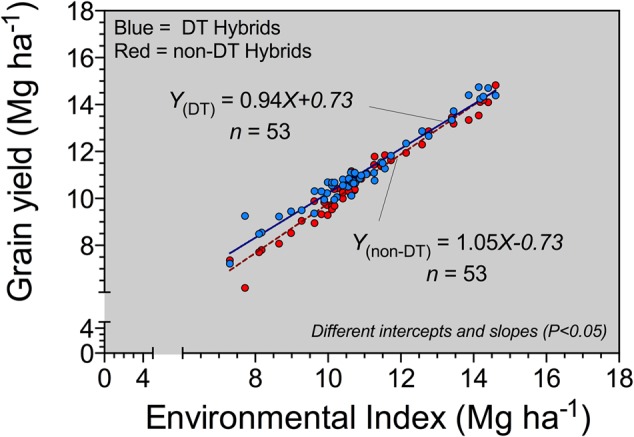
**Environmental index for drought-tolerant (DT, blue circles) and conventional or non-DT (non-DT, red circles) corn hybrids across several site-years in Kansas for the 2012 and 2013 growing seasons**.

As the environmental index decreased (associated with the most-limited water environments), DT hybrids had a better yield performance compared to non-DT hybrids (**Figure 2**). As resource availability, primarily connected to water supply, improved, DT and non-DT corn hybrid yield became more similar. A slope greater than 1 for the non-DT corn hybrid indicates above-average plasticity, and the slope less than 1 for the DT hybrid implies below-average plasticity. This agrees with the regression of DT hybrid yield advantage on non-DT hybrid yield (**Figure 1**) confirming that yields were similar for the two types of hybrids in environments supporting high yield levels, but DT hybrids performed better in environments supporting lower yields.

## Discussion

Historical genetic gain in corn was primarily associated with greater stress tolerance, mainly related to crowding stress (Duvick, 1999). Increasing plant density under optimal conditions can reduce plant growth rate and increase kernel abortion due to reduced resource availability on a per plant basis (water, nutrients, etc.). Drought tolerant technology in corn has been presented as a plant trait that can benefit crop yield production in water-limited environments (Castiglioni et al., 2008). The physiological mechanisms related to improvement in water utilization are still not clear; however, Nemali et al. (2015) suggested that a temporary reduction in leaf growth during periods of limited water reduced water use and stress perceived by the plant. They reported greater kernel set, harvest index, and grain yield for a hybrid with a DT trait compared to a control hybrid when water was limited. Tardieu (2013) reviewed the main effect of water shortage and its impacts on crops, highlighting the following three factors: (1) reduced growth of both vegetative and reproductive tissues, impacting leaf area, plant biomass, and silks in corn (Boyer, 1970); (2) stomatal closure, a genetically variable plant trait that can reduce water loss and minimize the impact of drought, but a consequent reduction of photosynthesis primarily affected reproductive organs when the stress occurred during the post-flowering period in corn (Zinselmeier et al., 1999); and (3) duration of the crop cycle, related to rapid senesce resulting from water shortage, with genetic variability documented in other crops, such as sorghum, associated with the stay-green trait (Borrell et al., 2000). Growth reduction, expressed as less leaf area, was previously quantified as a mechanism related to drought tolerance in corn, primarily connected to environments with severe terminal water shortage (Tardieu, 2012). However, the same author also emphasized that a mild stress with a large proportional reduction in growth could result in a reduction in yields. The severity and timing of growth reductions associated with limited water supply must balance reductions in plant water demand with maintaining adequate photosynthetic capacity and reproductive potential if yield is to be maintained or enhanced in water limited conditions.

Soil water conservation early during the growing season in field crops can produce a positive impact in water-limited environments by delaying water use for more critical reproductive stages. Diverse plant traits were identified as key factors that can play a critical role in better water use and therefore, drought tolerance capacity such as increased early-season water conservation (primarily via reduced early-season transpiration), limited-transpiration under high vapor pressure deficit (VPD), and rate of transpiration (focus early-season) when soil is drying (Gholipoor et al., 2012). For sorghum, the same authors documented a plateau-lineal association between transpiration rate and the fraction of transpirable soil water (FTSW), declining when FTSW values were less than 40%. For different corn hybrids, genotypic variation was reported for the association between transpiration and VPD (Gholipoor et al., 2013). Basically, two primary responses were documented: (1) continuous linear response, transpiration increases with VPD, and (2) a break point on transpiration with increasing VPD, presenting an advantage in water-limited environments with a potential of better early-season water conservation. In addition, Cooper et al. (2014) documented that DT hybrids show differential water consumption when compared with non-DT hybrids, reflecting higher water content at critical early and late reproductive stages for corn. The same authors reported a yield benefit when DT hybrids were compared with non-DT genotypes under both well- and limited-watered environments.

For the current study, the yield advantage documented for DT hybrids in high- and medium-ET environments supports the concept that DT hybrids enhance productivity in water-limited environments relative to non-DT hybrids. In an industry review study (2011–2013), Gaffney et al. (2015) documented an average three-fold greater yield benefit (6.5%) for DT corn hybrids in water-limited when compared with favorable environments (1.9%). The DT versus non-DT environmental index analysis, reflecting the complex interaction of all environments (E), genotypes (G), and management practices (M), G × E × M (Chapman, 2008; Sadras et al., 2009), displayed an expected positive relationship between yield and improvement in yield environment for both corn hybrid strategies. The non-DT hybrids improved their overall performance at a faster rate as the yield environment improved, associated in our data with low ET or full-IRRI regardless of ET. A plausible physiological explanation is connected to a lack of growth reduction by these hybrids under severe stress, resulting in greater plant biomass and transpiration and decreasing the efficiency of biomass allocation to the grain and negatively impacting yields relative to the DT hybrids. On the other hand, the DT hybrids showed less plasticity when compared with the non-DT counterparts. A larger yield gap, quantified as the yield difference between DT minus non-DT hybrids, resulted in poor yield environments. The latter is in agreement with results published by Roth et al. (2013) and Gaffney et al. (2015), both reflecting yield benefits for DT hybrids with limited water availability. There was minimal yield penalty for the DT hybrids in favorable environments, confirming that the DT hybrid did not sacrifice yield in low stress/high yield potential environments. The main limitation of our database used for the environmental analysis is that only nine different corn hybrids were evaluated, constraining the resolution of the outcomes presented in this study and potentially limiting the extent of inference. Notwithstanding the abovementioned limitation, all DT versus non-DT corn hybrid pairs followed a comparable response at varying yield environments, supporting the conclusions presented in this report and previous studies published on this topic (Roth et al., 2013; Gaffney et al., 2015).

## Conclusion

This data shows the greatest benefit from planting DT hybrids can be in environments with greater seasonal ET, with or without IRRI, and sites with smaller ET values if water becomes limiting within the season. The yield advantage of DT hybrids was positively correlated with environment ET, such that DT hybrids yielded more than non-DT hybrids in high and medium ET environments. In these situations, WUE for DT hybrids was significantly greater than for non-DT hybrids. Consequently, DT hybrids can offer some buffer against drought stress by reducing yield loss, but still offer adequate yield potential if drought is not a limiting factor for grain production. This makes DT hybrids a viable option in seasonally variable environments. The ability to offer some yield protection in higher stress environments, yet not sacrifice yield potential in low stress/high yield potential environments would be a benefit in areas where stress can vary considerably from season to season. Further research is needed to analyze the specific mechanisms of the DT hybrids that are involved in their response to drought stress. Improvements in drought tolerance should be further pursued when considering the drastic yield impact of drought at the national scale documented during the 2012 growing season (Boyer et al., 2013), reflecting the fragility of our corn production systems.

## Author Contributions

EA, KR, AS collected data, designed research, and performed research. EA, KR, AS, GB, and IC analyzed, and synthesized data, and wrote the paper.

## Conflict of Interest Statement

The authors declare that the research was conducted in the absence of any commercial or financial relationships that could be construed as a potential conflict of interest.

## Abbreviations

DT: drought tolerance
ET: evapotranspiration
non-DT: non-drought tolerant
SR: seeding rate
WUE: water use efficiency
